# Electrostatic-Interaction-Driven Assembly of Binary Hybrids towards Fire-Safe Epoxy Resin Nanocomposites

**DOI:** 10.3390/polym11020229

**Published:** 2019-02-01

**Authors:** Lu Liu, Wei Wang, Yongqian Shi, Libi Fu, Lulu Xu, Bin Yu

**Affiliations:** 1 College of Environment and Resources, Fuzhou University, 2 Xueyuan Road, Fuzhou 350116, China; lyqian@mail.ustc.edu.cn; 2 Hefei Institute for Public Safety Research, Tsinghua University, 5999 Xiyou Road, Hefei, Anhui 230026, China; 3 State Key Laboratory of Fire Science, University of Science and Technology of China, 96 Jinzhai Road, Anhui 230026, China; wwei433@mail.ustc.edu; 4 Department of Architecture and Civil Engineering, City University of Hong Kong, Tat Chee Avenue, Kowloon 999077, Hong Kong; 5 College of Civil Engineering, Fuzhou University, 2 Xueyuan Road, Fuzhou 350116, China; fulibi@fzu.edu.cn; 6 School of Materials Science & Engineering, Nanyang Technological University, Singapore 639798, Singapore; lulu.xu@ntu.edu.sg

**Keywords:** binary hybrids, manganese dioxide, zinc hydroxystannate, flame retardancy, epoxy resin

## Abstract

Manganese dioxide (MnO_2_), as a promising green material, has recently attracted considerable attention of researchers from various fields. In this work, a facile method was introduced to prepare binary hybrids by fabricating three-dimensional (3D) zinc hydroxystannate (ZHS) cubes on two-dimensional (2D) MnO_2_ nanosheets towards excellent flame retardancy and toxic effluent elimination of epoxy (EP) resin. Microstructural analysis confirmed that the morphologies and structures of MnO_2_@ZHS binary hybrids were well characterized, implying the successful synthesis. Additionally, the morphological characterization indicated that MnO_2_@ZHS binary hybrids could achieve satisfactory interfacial interaction with the EP matrix and be well dispersed in nanocomposites. Cone calorimeter test suggested that MnO_2_@ZHS binary hybrids effectively suppressed the peak of heat release rate and total heat release of EP nanocomposites, performing better than MnO_2_ or ZHS alone. Condensed-phase analysis revealed that MnO_2_@ZHS binary hybrids could promote the char density and graphitization degree of char residues and thereby successfully retard the permeation of oxygen and flammable gases. Moreover, through the analysis of gas phase, it can be concluded that MnO_2_@ZHS binary hybrids could efficiently suppress the production of toxic gases during the degradation of EP nanocomposites. This work implies that the construction of 2D/3D binary hybrids with an interfacial interaction is an effective way to fabricate high-performance flame retardants for EP.

## 1. Introduction

Epoxy resin (EP), one of the most important thermosetting polymers, has been widely used in the fields of furniture coatings [1], circuit boarding [2], aeronautical material [3], and so forth [4,5,6] in virtue of its excellent mechanical properties, outstanding optical property, glorious resistance to solvent and chemical corrosion, and high climate resistance. However, analogous to most other polymers, EP resin can be easily ignited and sharply spread, accompanied with large amounts of smoke and heat release. It is well known that smoke and toxic gases are the main reasons for death in fire accidents. Hence, to meet fire safety regulations and expand the use of EP in the fields that require flame resistance, a significant number of flame-retardant techniques for EP resins are developed. Some kinds of flame retardants have been gradually forbidden in most countries, due to toxic gases, such as hydrogen cyanide, contained in the pyrolysis products during the combustion process [7,8]. For instance, the most used brominated flame retardants often result in the formation of notorious pollutants, most notably, brominated and mixed halogenated dioxins [9,10]. Therefore, how to prepare green and effective flame retardants for EP remains a global topic for researchers.

In recent years, the nanoscale fillers, such as inorganic materials, have attracted considerable attention to impart polymers with excellent mechanical properties, antibacterial properties, flame retardancy, and so forth [11,12,13,14,15,16]. Generally, inorganic materials can endow polymers with excellent flame retardancy at a low loading. Moreover, different from other organic flame retardants, the incorporation of inorganic materials with a low addition will not deteriorate the mechanical properties of polymer materials. Inorganic materials with different morphologies and element composition show total flame retardancy mechanism towards a variety of polymers. Usually, one-dimensional inorganic materials, such as carbon nanotubes, FeOOH, and titanium dioxides [17,18,19,20,21], could form networks during the degradation process of the polymer matrix, which can play the function of physical barrier retarding the permeation of heat, oxygen, and pyrolysis products. Two-dimensional (2D) materials, including graphene, layered double hydroxides, molybdenum disulfide, and manganese dioxide (MnO_2_), are usually regarded as the most efficient in improving the flame retardancy of polymers in virtue of their large surface area [22,23,24], which can effectively delay the transfer of oxygen and toxic and flammable gases. For the three-dimensional materials, for instance, silica spheres, titanium dioxides spheres, and zinc hydroxystannate (ZHS) [25,26,27], these inorganic materials can also improve the flame retardancy of polymers; the catalytic charring effect and large inner specific surface area are the main reasons for the enhanced properties. It is reported that the Lewis acid catalyst forms an initial cation, promoting the Friedel–Crafts reaction, which can effectively build C–C bonds with aromatic compounds, thereby improving the char density [28]. In the case of ZHS cubes, such as metal hydroxides, they have been regarded as affinity probes, which possess the Lewis acidity [29]. Therefore, ZHS cubes are usually used as an efficient flame retardant to promote the formation of char residue.

Up to date, the concept of multicomponent systems has been applied in a variety of fields, including environment, lithium batteries, water research, and materials. In the case of materials, multicomponent systems can be realized by different methods. Very recently, the layer-by-layer technique has been employed to improve the flame retardancy of polymers such as cotton, polyamides, and flexible polyurethane foam [30,31]. This technique is mainly driven by the forces from electrostatic interactions, hydrogen bond interactions, and covalence bonds. A wide variety of functional molecules can be incorporated within the layer-by-layer coating, including polyelectrolytes and inorganic materials, to impart the matrix with the desired properties.

In this work, a binary component consisting of 2D MnO_2_ nanosheets and three-dimensional (3D) ZHS binary hybrids was synthesized by the self-assembly method to endow the EP matrix with flame retardancy and toxic suppression properties. In our previous works, MnO_2_ nanosheets were confirmed to have an excellent physical barrier effect during the degradation process in virtue of their two-dimensional structure [32,33]. Hence, fabrication of 2D/3D MnO_2_@ZHS binary hybrids was anticipated to achieve excellent flame retardancy and toxic effluent elimination, and the corresponding flame retardancy mechanism was also explored.

## 2. Materials and Methods

### 2.1. Raw Materials

All chemicals were of analytical grade and used as received without further purification. Zinc sulfate heptahydrate (ZnSO_4_·7H_2_O), sodium stannate trihydrate (Na_2_SnO_3_·3H_2_O), and potassium permanganate (KMnO_4_) were supplied by Sinopharm Chemical Reagent Co. Ltd. (Shanghai, China). Bisphenol-A type EP was purchased from Shixian Chemical Industry Co., Ltd. A curing agent (diaminodiphenylmethane) and sodium hydroxide were purchased from Sinopharm Chemical Reagent Co., Ltd.

### 2.2. Synthesis of MnO_2_ Nanosheets and ZHS Cubes

The synthesis process of MnO_2_ was according to the reported work [34]. In a typical process, 15 mmol of KMnO_4_ was completely dissolved in 750 mL of deionized water. Then, 200 mL of ethyl acetate was added into the above solution by constantly stirring. The solution was maintained at 95 °C and refluxed overnight. The obtained brown product was filtrated and washed with deionized water and ethanol consecutively, then dried at 80 °C for further use. In the case of ZHS cubes [25], 10 mmol of ZnSO_4_·7H_2_O was dissolved in 300 mL of deionized water at 25 °C. Once completely dissolved, the equal loading of Na_2_SnO_3_·H_2_O was added into the above solution. The continuous stirring was maintained for 4 h, and the white suspension was centrifuged and washed with deionized water several times and then dried at 60 °C overnight.

#### 2.2.1. Preparation of MnO_2_@ZHS Binary Hybrid

MnO_2_@ZHS binary hybrid was prepared by a simple electrostatic adsorption method. MnO_2_ (1 g) was dispersed in 500 mL of deionized water with ultrasound-assisted mechanical agitation for 2 h. On the other hand, 0.2 g of ZHS was dispersed in 300 mL of deionized water under ultrasonic and mechanical stirring for 1 h. The pH value of ZHS suspension was adjusted to 5 by 0.1 M HCl solution and stirred for another 1 h. Subsequently, the ZHS suspension was dropwise added into the MnO_2_ suspension within 30 h by constant mechanical stirring. Finally, the mixed suspension was stirred and maintained for 4 h. The resultant was selected by centrifugation, washed by deionized water and dried at 80 °C overnight.

#### 2.2.2. Preparation of Pure EP and EP Nanocomposites

**Figure 1:**
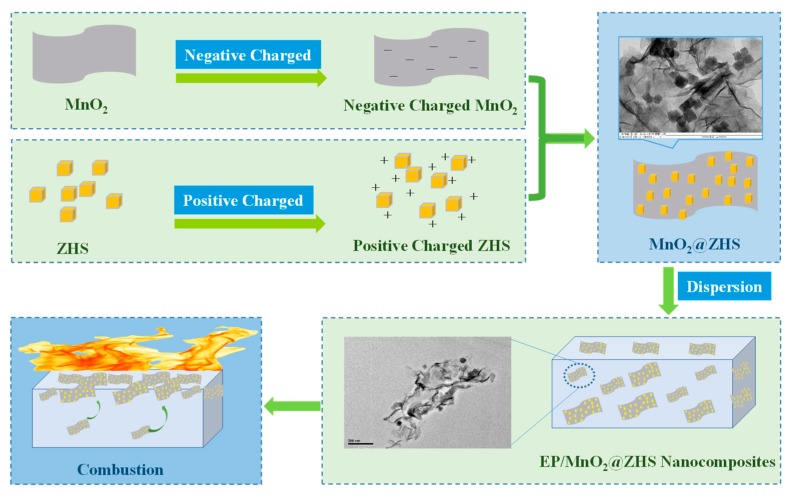
Synthesis of MnO_2_@ZHS hybrids and flame retardancy mechanism in epoxy.

The preparation of EP nanocomposites was in accordance with our previous works [18]. Typically, 1.22 g of MnO_2_@ZHS binary hybrid was added into 50 mL of acetone under ultrasonically assisted stirring to form a homogeneous suspension. Then, 50 g of premelted EP was poured into the above suspension under mechanical stirring and maintained over 6 h. Subsequently, the suspension was maintained at 100 °C for 12 h to remove the solvent. Thereafter, 10 g of premelted 4,4’-diaminodiphenylmethane was injected into the mixture under constant stirring. Finally, EP nanocomposites could be obtained after being cured at 100 °C for 2 h and 150 °C for 2 h, respectively. The sample was marked as EP/MnO_2_@ZHS 2%, and the others were fabricated by using the same strategy. The preparation process is illustrated in Figure 1.

### 2.3. Characterization

X-ray diffraction (XRD) measurements were performed using a Japan Rigaku D = Max-Ra rotating anode X-ray diffractometer (RIGAKU, Tokyo, Japan) equipped with a Cu–Ka tube and Ni filter (k = 0.1542 nm). Transmission electron microscopy (TEM) images were obtained using a JEOL JEM-100SX transmission electron microscope (JEOL, Tokyo, Japan) with an acceleration voltage of 100 kV. Thermogravimetric analysis (TGA) of as-prepared samples was undertaken using TGA-Q5000 apparatus (TA Co., New Castle, DE, USA) from 50 to 700 °C at a heating rate of 20 °C min^−1^. The weight of all samples was maintained within 3–5 mg in an open platinum pan. The morphologies of MnO_2_ materials with three different dimensions, coated with a gold layer in advance, were observed using scanning electron microscopy (SEM; AMRAY1000B, Beijing R&D Center of the Chinese Academy of Sciences, Beijing, China). TG-IR of the samples was performed using a TGA Q5000IR thermal gravimetric analyzer (TA Co., New Castle, DE, USA) that was interfaced to the Nicolet 6700 FTIR spectrophotometer (Thermo Scientific, Waltham, MA, USA). Approximately 5.0 mg of the sample was placed in an alumina crucible and heated from 30 to 800 °C at a heating rate of 20 °C min^−1^ (helium atmosphere, flow rate of 45 mL min^−1^). A combustion test was performed on the cone calorimeter (Fire Testing Technology, Derby, UK) according to ISO 5660 standard, using specimens with size of 100 × 100 × 3 mm^3^. Each specimen was exposed horizontally to an external heat flux of 35 kW m^−2^. X-ray photoelectron spectroscopy (XPS) was performed on a VG Escalab Mark II spectrometer (Thermo-VG Scientific Ltd., West Sussex, UK), using Al Kα excitation radiation (hυ = 1486.6 eV).

## 3. Results and Discussion

### 3.1. Characterization of MnO_2_@ZHS Binary Hybrid

**Figure 2:**
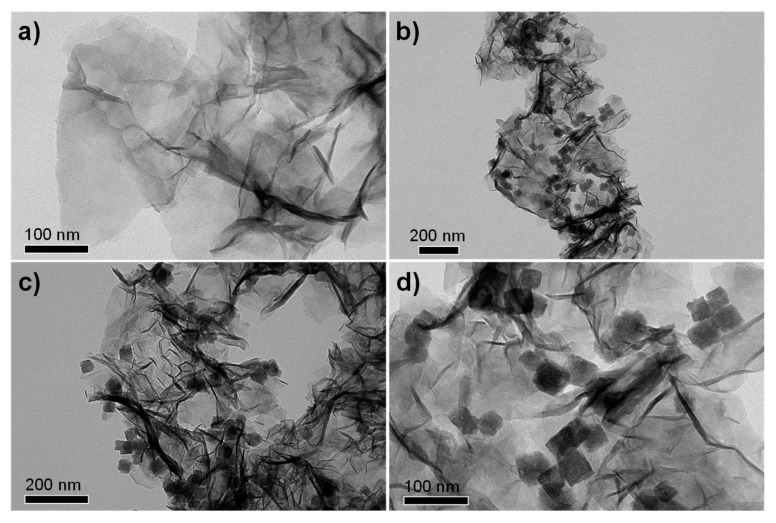
TEM images of (**a**) MnO_2_ nanosheets, and (**b**–**d**) MnO_2_@ZHS hybrid.

**Figure 3:**
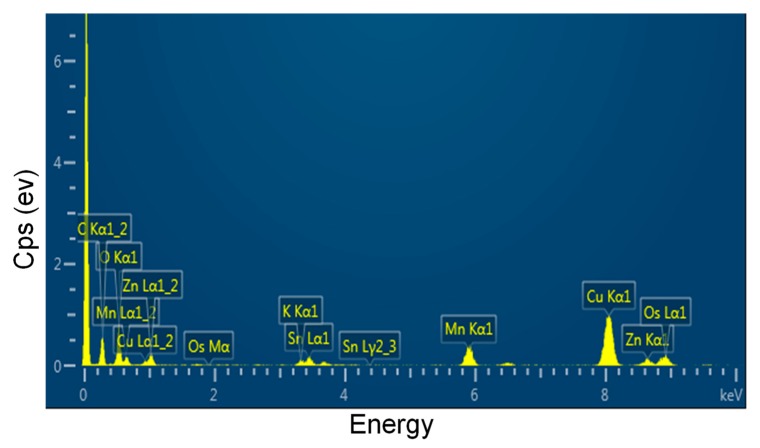
EDS profile of MnO_2_@ZHS.

**Figure 4:**
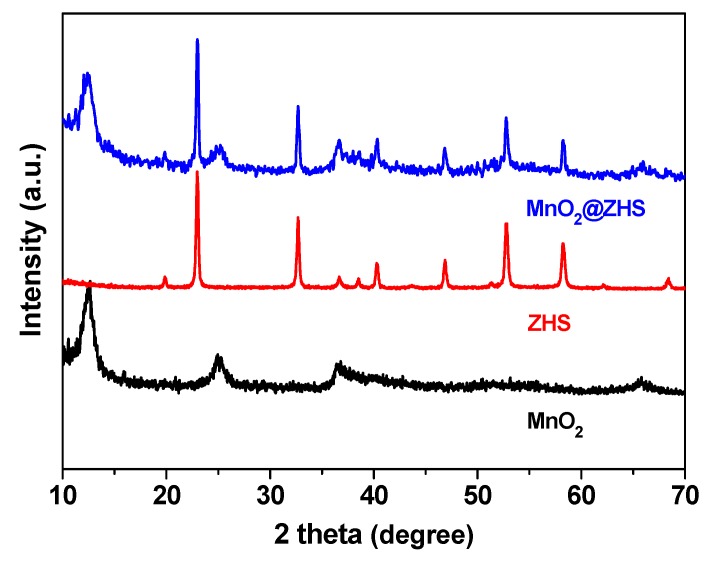
XRD patterns of MnO_2_, ZHS, and MnO_2_@ZHS hybrid.

**Figure 5:**
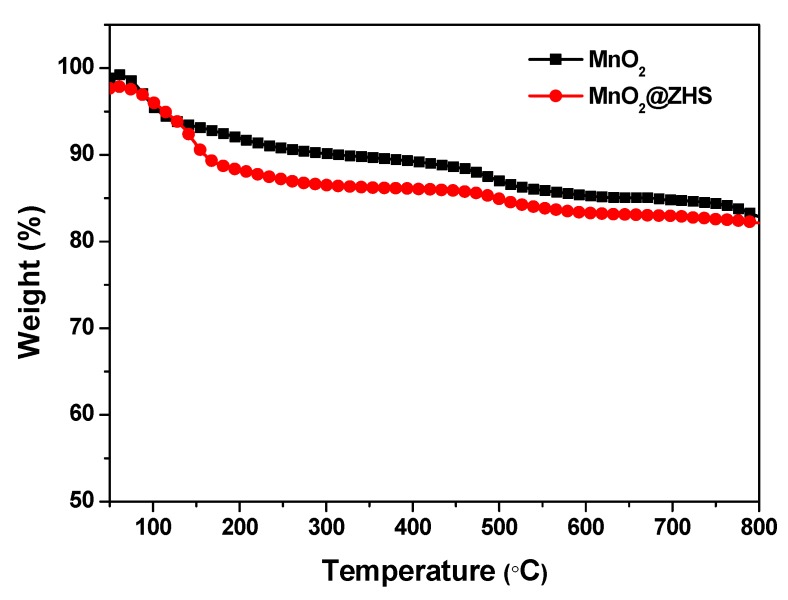
TGA curves of MnO_2_ and MnO_2_@ZHS.

TEM technique was employed to observe the morphologies of the MnO_2_ nanosheets and the MnO_2_@ZHS binary hybrid, and the morphologies of the MnO_2_ nanosheets and their hybrid are presented in Figure 2. As can be observed in Figure 2a, the MnO_2_ nanosheets show layered structure with large surface area, and the wrinkle belonging to the layers can be obviously observed. After the assembly procedure forced by electrostatic adsorption, ZHS cubes are successfully fabricated on the surface of MnO_2_. As is shown in Figure 2b–d, the ZHS cubes are firmly absorbed on the layers, and no ZHS cubes exist in the area beside the MnO_2_ nanosheets, indicating that there exist very strong electrostatic forces between the ZHS cubes and the MnO_2_ nanosheets. Moreover, the EDS spectrum of the MnO_2_@ZHS binary hybrid is presented in Figure 3. The EDS profile confirms the presence of corresponding elements such as Zn, Sn, Mn, and O. Figure 4 presents XRD patterns of the MnO_2_ nanosheets, ZHS cubes, and the MnO_2_@ZHS binary hybrid. It is evident that the MnO_2_@ZHS binary hybrid contains all the characteristic peaks of MnO_2_ and ZHS [35,36]. These results indicate that the crystalline structures of ZHS and MnO_2_ have no changes during the absorption procedure. Moreover, TGA was used to measure the thermal stability of the MnO_2_@ZHS binary hybrid and investigate the changes compared with that of the MnO_2_ nanosheets. Figure 5 shows the TGA curves of the MnO_2_ nanosheets and MnO_2_@ZHS binary hybrid. The thermal stability of the MnO_2_@ZHS binary hybrid is relatively lower than that of MnO_2_ nanosheets, due to the existence of predecomposition compared with MnO_2_. It is probably because of the separation of hydrate water from ZHS [37]. The further chemical state and element composition of MnO_2_ and MnO_2_@ZHS were explored by XPS test.

**Figure 6:**
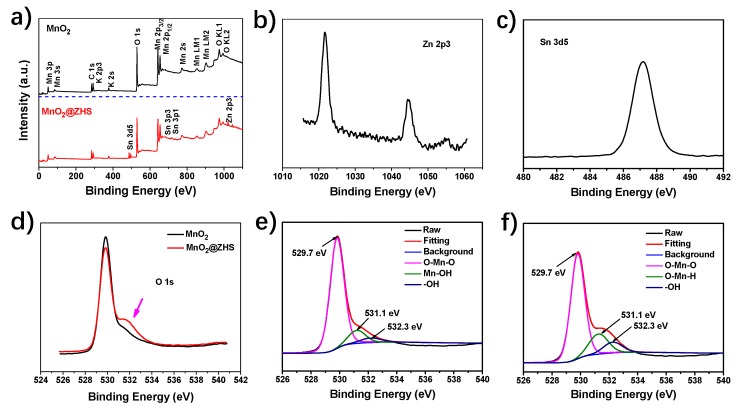
(**a**) XPS survey spectra of MnO_2_ and MnO_2_@ZHS binary hybrid, (**b**) the high-resolution Zn 2p3, and (**c**) Sn 3d5 spectra of MnO_2_@ZHS binary hybrid, (**d**) O1s spectra of MnO_2_ and MnO_2_@ZHS binary hybrid, the deconvoluted O1s spectra of (**e**) MnO_2_ and (**f**) MnO_2_@ZHS binary hybrid.

In Figure 6a, the XPS survey spectra of MnO_2_ and MnO_2_@ZHS are presented. The XPS survey spectra indicate that the MnO_2_@ZHS hybrid is mainly comprised of Mn, O, Sn, Zn, K, and C elements. The high-resolution Zn 2p^3^ and Sn 3d are also shown in Figure 6b,c. The binding energies of Zn 2p^3^ and Sn 3d are centered at 121.5 and 486.7 eV, respectively, which are attributed to Zn–O and Sn–O bonds [38]. Additionally, the high-resolution O1s spectra is exhibited in Figure 6d–f. The location at 529.8 eV is ascribed to the O–Mn–O bond, and the peak centered at 531.2 eV belongs to the O–Mn–H bond [39,40]. Additionally, it can be clearly found that the binding energy of O1s at around 532.3 eV attributed to the weak bond (-OH) of the MnO_2_@ZHS binary hybrid is higher than that of MnO_2_. This is reasonable due to the fabrication of ZHS, which is accompanied by abundant hydroxyl groups.

### 3.2. Interfacial Adhesion Beween MnO_2_@ZHS Binary Hybrid and EP Matrix

**Figure 7:**
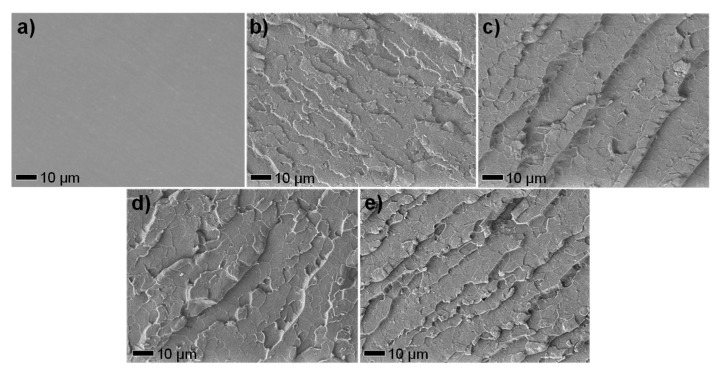
SEM images of fracture surfaces cryogenically broken after immersion in liquid nitrogen of (**a**) pure EP, (**b**) EP/MnO_2_ 2%, (**c**) EP/MnO_2_@ZHS 0.5%, (**d**) EP/MnO_2_@ZHS 1%, and **e)** EP/MnO_2_@ZHS 2%.

**Figure 8:**
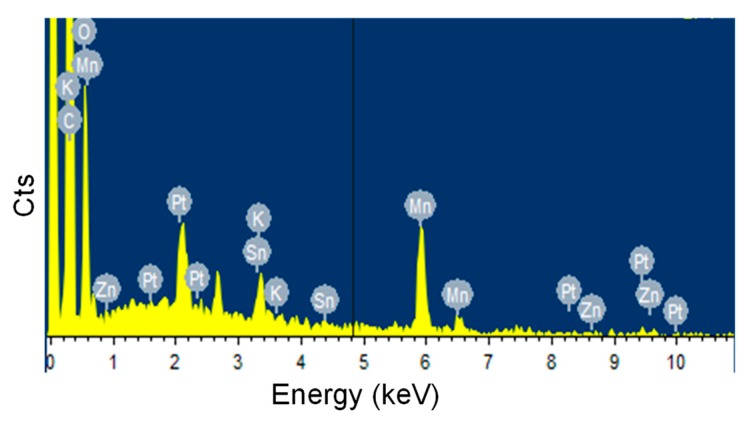
EDS profile of EP/MnO_2_@ZHS 2%.

The morphology of the fracture surface of the samples is observed by SEM test, in order to further investigate the interfacial interaction between the nanofillers and EP matrix. As can be observed in Figure 7a, the fracture surface of pure EP shows smooth and clean. After the addition of MnO_2_ nanosheets, the surface of EP/MnO_2_ with a 2 wt % loading appears rough and wrinkled, indicating the improved interfacial interaction between the MnO_2_ nanosheets and EP matrix (Figure 7b). In the case of EP/MnO_2_@ZHS with different loadings, the wrinkles on the surface show deeper than the addition of MnO_2_, declaring that the interfacial interactions between the MnO_2_@ZHS binary hybrid and EP matrix are improved (Figure 7c–e). Moreover, as the loading of the MnO_2_@ZHS binary hybrid increases, the fish-like folds become more delicate. EP/MnO_2_@ZHS 2% exhibits the most delicate surface among the samples, which is reasonably due to the increase of MnO_2_@ZHS acting as stress points. Moreover, the EDS information of Figure 7e is listed in Figure 8, and the elements from the MnO_2_@ZHS binary hybrid can also be found. Therefore, the results obtained from SEM images demonstrate that the incorporation of MnO_2_@ZHS can improve the interfacial interaction between MnO_2_@ZHS and the EP matrix.

### 3.3. Thermal Stabilities of Pure EP and Its Nanocomposites Studied by TGA Test

**Figure 9:**
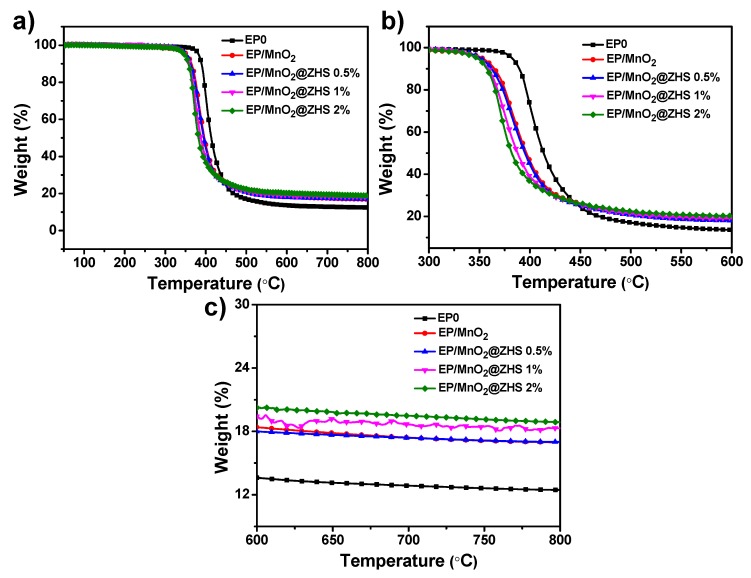
(**a**) TGA curve of pure EP and EP nanocomposites under nitrogen condition. (**b**,**c**) represent magnified TGA profiles of (**a**) at different temperatures.

The thermal stability of pure EP and EP nanocomposites is studied by TGA test, as shown in Figure 9. The onset decomposition temperature and the temperature at the maximal weight loss rate are denoted as T_-10_ and T_max_, respectively. Pure EP and EP nanocomposites exhibit the same degradation process, showing a one-stage degradation process, which is ascribed to the decomposition of macromolecular chains. Additionally, it can be seen that after the incorporation of MnO_2_, the char residue of EP/MnO_2_ at 800 °C increases compared with that of pure EP, but the T_-10%_ and T_max_ of EP/MnO_2_ decrease, which is possibly attributed to the catalysis effect from MnO_2_ promoting the predecomposition of the matrix and the degradation of metal oxides. In the case of EP/MnO_2_@ZHS nanocomposites, the values of T_-10%_ and T_max_ further decrease, which can be due to the predegradation of ZHS, thereby resulting in the predecomposition of the matrix. However, the increased residual content at 800 °C of EP/MnO_2_@ZHS nanocomposites is enhanced with the increase of the loading. Therefore, the addition of the MnO_2_@ZHS binary hybrid improves the char residue of the EP matrix.

### 3.4. Flame Retardancy Evaluated by Cone Calorimeter

**Figure 10:**
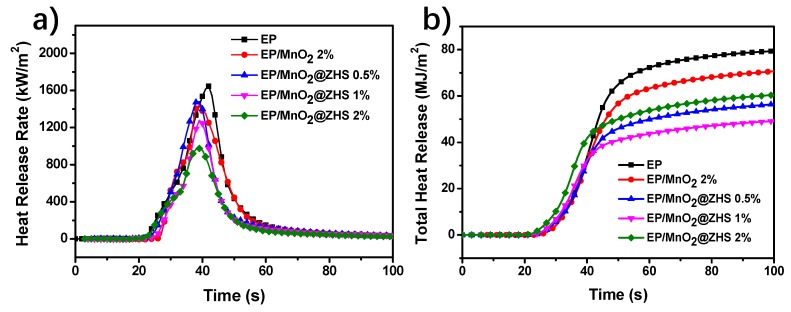
(**a**) HRR and (**b**) THR curves of pure EP and EP nanocomposites evaluated by cone calorimeter.

The cone calorimeter is generally regarded as the most powerful tool to evaluate the force burning fire performance of materials through simulating a developing fire scenario on a fixed-sized specimen. The parameters, including peak heat release rate (PHRR) and total heat release (THR), are recognized as the crucial data for evaluating the fire safety of matrix. HRR and THR curves of pure EP and EP nanocomposites are presented in Figure 10. According to Figure 10a, incorporating MnO_2_ into the EP matrix can reduce the values of PHRR of EP nanocomposites, which is mainly attributed to the physical barrier effect. In the case of EP/MnO_2_@ZHS nanocomposites, all the values of PHRR are decreased as compared to that of pure EP. Moreover, as the amount of percent add-ons increases, the value of PHRR is further reduced. Thus, in terms of the decreased PHRR values, it can be indicated that the MnO_2_@ZHS hybrid shows the superior inhibition of heat release of the EP matrix. In addition, THR values of all EP nanocomposites shown in Figure 10b decrease obviously, in comparison with the pure one, showing that both MnO_2_ and the MnO_2_@ZHS binary hybrid can effectively suppress the heat release of EP nanocomposites during combustion. Moreover, the THR value of EP/MnO_2_@ZHS with a 2 wt % loading can achieve near 40% reduction, compared with the control EP, which is rarely attained for the nanocomposites. The results of cone calorimetry illustrate that the function of charring formation of ZHS is successfully grafted on the MnO_2_ nanosheets. The catalytic charring function, combined with the physical barrier effect from the MnO_2_ nanosheets, can significantly enhance the flame retardancy of EP nanocomposites.

### 3.5. Char Residues Analysis of EP and EP/MnO_2_@ZHS Nanocomposites

**Figure 11:**
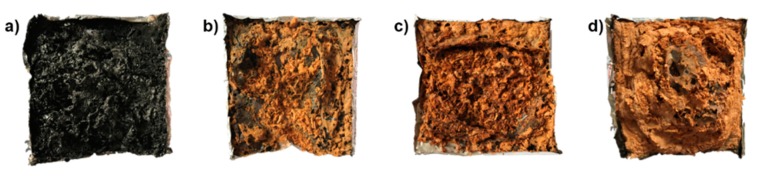
Digital images of char residues for (**a**) pure epoxy, (**b**) EP/MnO_2_@ZHS 0.5%, (**c**) EP/MnO_2_@ZHS 1%, (**d**) EP/MnO_2_@ZHS 2% after cone calorimeter tests. The scale of these samples is 100 × 100 mm^2^.

Digital photos of char residues of pure EP and EP nanocomposites are plotted in Figure 11, in order to directly observe their morphologies. As shown in Figure 11a, pure EP exhibits lower amount of char residues compared with other EP nanocomposites incorporated by MnO_2_@ZHS binary hybrids. According to Figure 11d, the char residue of EP/MnO_2_@ZHS 2% shows a larger volume, illustrating a more effective catalytic charring formation by MnO_2_@ZHS. Additionally, the char residues of EP nanocomposites are covered by yellow-colored materials, which are the pyrolysis products obtained by the degradation of the MnO_2_@ZHS binary hybrids. Usually, for inorganic nanocomposites, the inorganic fillers would transfer to the surface of the matrix during the decomposition process. These yellow-colored degradation products can act as the insulation barrier, delaying the permeation of oxygen and flammable gases, thereby reducing the values of both PHRR and THR.

**Figure 12:**
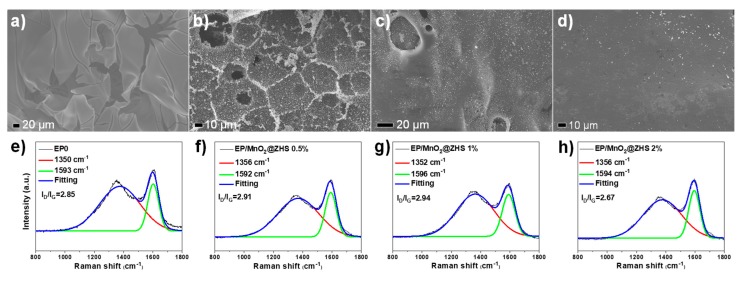
SEM images and Raman spectra of the char residues of (**a**,**e**) pure EP, (**b**,**f**) EP/MnO_2_@ZHS 0.5%, (**c**,**g**) EP/MnO_2_@ZHS 1%, and (**d**,**h**) EP/MnO_2_@ZHS 2%.

To investigate the surface morphologies of char residues of pure EP and EP/MnO_2_@ZHS, the char residues studied here were obtained after cone calorimetry and imaged via SEM test. According to the char residue of pure EP portrayed in Figure 12a, deep cracks and collapses are obviously observed and thereby lead the oxygen and flammable gases to transfer easily. After the incorporation of MnO_2_@ZHS into the EP matrix, the char residue shows as more intact with less cracks (Figure 12b–d). Furthermore, as the loading increases up to 2 wt %, the surface of EP/MnO_2_@ZHS is very smooth, and there are no collapses and cracks, which can perfectly retard the permeation of oxygen and toxic and flammable products. Overall, SEM images visually confirm that the addition of MnO_2_@ZHS can significantly improve and perfect the char formation of EP nanocomposites. Besides, the Raman test was used to further study the inner graphitic structure of the char residues. As shown in Figure 12e–h, the Raman spectra of pure EP and EP nanocomposites all present two visible characteristic bands, namely, the D-band (1353 cm^−1^) and G-band (1592 cm^−1^), respectively attributed to the vibration of carbon atoms with dangling bonds in the plane terminations of disordered graphite or glass carbons and the vibration of sp^2^-bond carbon atoms in graphite layers. Generally, the ratio of the intensity of the D-band and G-band (I_D_/I_G_) is used to evaluate the graphitization degree of char residues. It is well recognized that sp^2^-bond carbon atoms in graphite layers can behave as insulation barriers retarding the transfer of heat, oxygen, and flammable products. For the value of I_D_/I_G_, the lower value means the better graphitic structure and physical insulation [41]. With the incorporation of MnO_2_@ZHS in the EP matrix, the value of I_D_/I_G_ gradually becomes lower. As the loading increases, EP/MnO_2_@ZHS 2% exhibits the lowest value of I_D_/I_G_, compared with the others, indicating the most intact char structure. These results are in good agreement with the SEM results.

**Figure 13:**
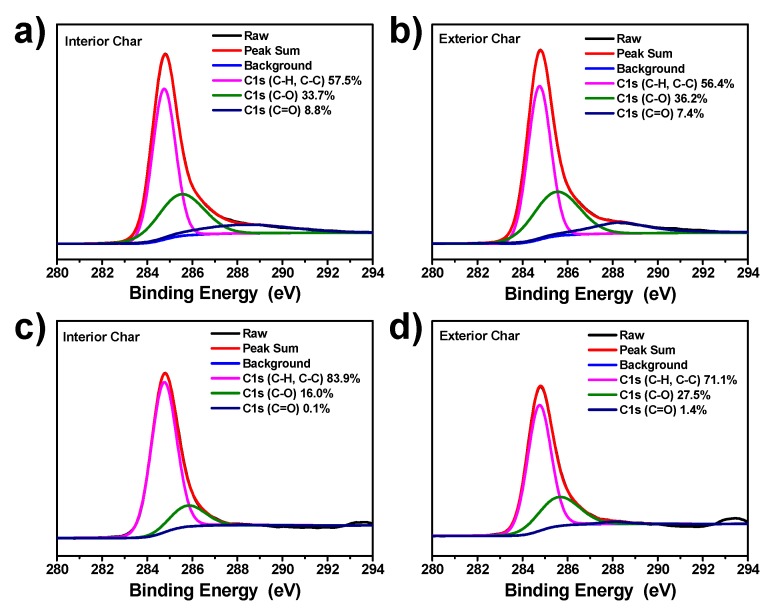
The high-resolution C1s spectra of interior and exterior char residues of (**a**,**b**) pure EP and (**c**,**d**) EP/MnO_2_@ZHS 2%.

The XPS test was employed to further explore the carbon structure of interior and exterior char residues of pure EP and EP/MnO_2_@ZHS 2% and their flame retardancy mechanism, as plotted in Figure 13. For all the high-resolution C 1s spectra, it can be deconvoluted to three characteristic peaks centered at 284.6, 285.6, and 288.3 eV, assigned to aliphatic and aromatic species, hydroxyl group and/or ether, and carbonyl groups, respectively. It is generally accepted that the ratios of C_ox_ /C_a_ (C_ox_: oxidized carbon; C_a_: aliphatic and aromatic carbons) are employed to evaluate the thermal oxidative resistance of EP nanocomposites [18]. The lower value of C_ox_ /C_a_ means better thermal oxidative resistance. It is found that the values of C_ox_ /C_a_ in interior and exterior char residues of pure EP are 0.74 and 0.77, respectively, which are much higher than those of EP/MnO_2_@ZHS 2% (0.19 and 0.41). Therefore, based on these results, it can be well confirmed that MnO_2_@ZHS can promote the char density and graphitization degree of char residues, thereby efficiently enhancing the flame retardancy of the EP matrix.

### 3.6. Gas-Phase Analysis of Pure EP and EP Nanocomposites

**Figure 14:**
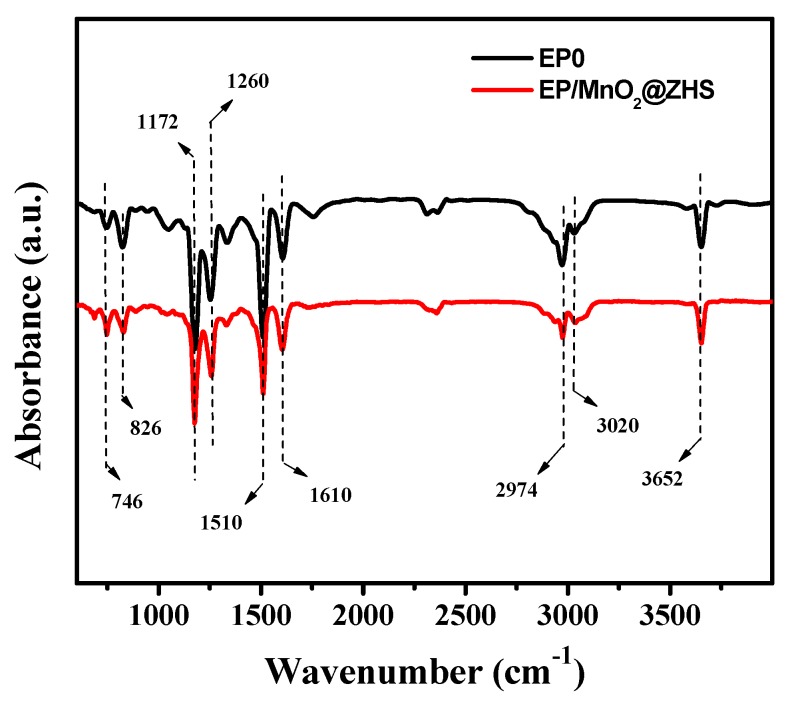
FT-IR spectrum of pyrolysis products of pure EP and EP/MnO_2_@ZHS 2% composites at the maximum decomposition rate.

It is well known that the pyrolysis products of the EP matrix are extremely harmful to the health of humans. Here, TG-IR technique was adopted to study the composition of pyrolysis products during the degradation process of the EP matrix and further investigate the gas-phase mechanism. The FTIR spectra of the pyrolysis products of pristine EP and EP/MnO_2_@ZHS 2% are presented in Figure 14. It can be concluded that the composition of the pyrolysis products is rarely changed, and the characteristic peaks are strictly ascribed to the functional groups with unambiguous band positions, such as alkane groups mainly for allyl alcohol; hydrocarbons (2820–3120 m^−1^); aromatic compounds (750, 830, 1460, 1510, and 1610 cm^−1^); CO (2180 cm^−1^); CO_2_ (2356 cm^−1^); and water and/or phenol (ca. 3640–3670 cm^−1^) [26,33].

**Figure 15:**
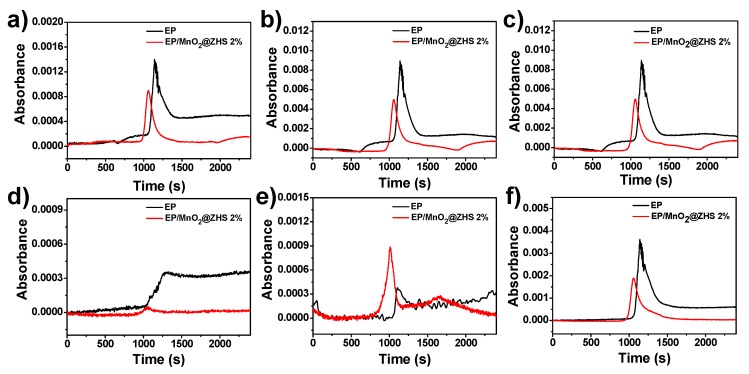
Absorbance of pyrolysis products versus time for EP and its nanocomposites: (**a**) Gram–Schmidt; (**b**,**c**) aromatic compounds located at 1510 and 1610 cm^−1^; (**d**) CO; (**e**) CO_2_; and (**f**) hydrocarbons.

**Figure 16:**
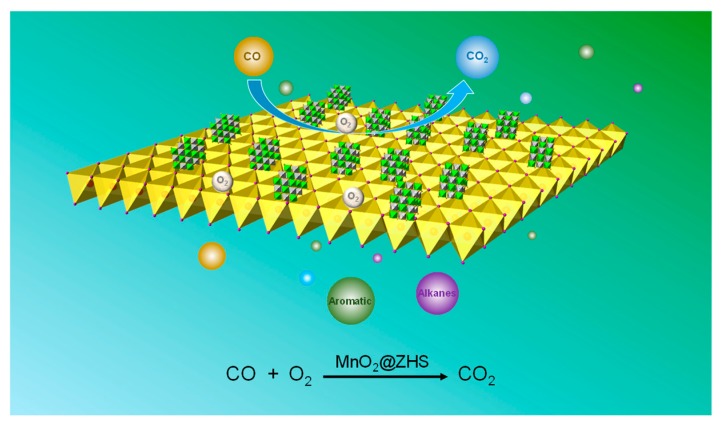
The proposed mechanism of catalytic conversion of CO into CO_2_.

Figure 15a shows the Gram–Schmidt curves of EP and EP/MnO_2_@ZHS 2%. The incorporation of MnO_2_@ZHS binary hybrids obviously reduces the absorbance of pyrolysis products, rivalled by that of pure EP. Moreover, Figure 15b,c,f suggest that aromatic compounds and alkane groups are all effectively decreased, implying the reduction of organic gases and improved toxic effluent elimination. Additionally, Figure 15d,e respectively present the absorbance of CO and CO_2_, and it can be seen that as the absorbance of CO decreases, the absorbance of CO_2_ increases for all the nanocomposites. This could not have an alternative explanation that less pyrolysis gases penetrate the char, making the combustion more ventilated. Therefore, it might very well be a catalysis effect for conversion of CO into CO_2_. In summary, TG-IR results demonstrate that MnO_2_@ZHS binary hybrids can efficiently suppress the production of toxic gases during the degradation of EP nanocomposites, and the addition of binary hybrids can catalyze conversion of CO into CO_2_. The mechanism of catalytic conversion of CO into CO_2_ is proposed in Figure 16. Combined with the XPS results obtained from Figure 6, it is inferred that the capacity of Mn to adapt several oxidation states enables it to undergo oxygen reduction reactions, indicating MnO_2_ nanosheets can act as a reservoir for oxygen, analogous to the previous work [42]. Certainly, toxic gases in real scenarios only contain CO instead of a wide array of hydrocarbons. Therefore, the catalytic conversion of CO into CO_2_ is of great significance. In addition, the increase of CO_2_ can dilute the oxygen and flammable gases in the air and thereby retard the combustion process.

## 4. Conclusions

In this work, a facile method was introduced to synthesize binary hybrids by fabricating ZHS cubes on MnO_2_ nanosheets with better flame retardancy and toxic effluent elimination of EP. XRD, TEM, and XPS results confirmed the successful synthesis of MnO_2_@ZHS binary hybrids. Moreover, the SEM and TEM ultrathin images indicated that MnO_2_@ZHS binary hybrids could achieve strong interfacial interaction with the EP matrix and be well dispersed in nanocomposites. Cone calorimeter test suggested that MnO_2_@ZHS binary hybrids could effectively suppress the curves of HRR and THR of EP nanocomposites, performing better than MnO_2_ or ZHS alone. Condensed-phase analysis revealed that MnO_2_@ZHS binary hybrids could promote the char density and graphitization degree of char residues and thereby successfully retard the permeation of oxygen and flammable gases. Additionally, it was concluded that MnO_2_@ZHS binary hybrids could efficiently suppress the production of toxic gases during the degradation of EP nanocomposites through the analysis of gas phase.

## References

[1] Wu G.M., Kong Z.W., Chen J., Huo S.P., Liu G.F. (2014). Preparation and properties of waterborne polyurethane/epoxy resin composite coating from anionic terpene-based polyol dispersion. Prog. Org. Coat..

[2] Hadi P., Xu M., Lin C.S., Hui C.W., McKay G. (2015). Waste printed circuit board recycling techniques and product utilization. J. Hazard. Mater..

[3] Zhang H., Wang P., Yang J. (2014). Self-healing epoxy via epoxy–amine chemistry in dual hollow glass bubbles. Compos. Sci. Technol..

[4] Guadagno L., Raimondo M., Vittoria V., Vertuccio L., Naddeo C., Russo S., De Vivo B., Lamberti P., Spinelli G., Tucci V. (2014). Development of epoxy mixtures for application in aeronautics and aerospace. RSC Adv..

[5] Vietri U., Guadagno L., Raimondo M., Vertuccio L., Lafdi K. (2014). Nanofilled epoxy adhesive for structural aeronautic materials. Compos. Part B-Eng..

[6] Shi Y.Q., Yu B., Zheng Y.Y., Guo J., Chen B.H., Pan Z.M., Hu Y. (2018). A combination of POSS and polyphosphazene for reducing fire hazards of epoxy resin. Polym. Adv. Technol..

[7] Watanabe I., Sakai S.I. (2003). Environmental release and behavior of brominated flame retardants. Environ. Int..

[8] Czégény Z., Jakab E., Blazsó M., Bhaskar T., Sakata Y. (2012). Thermal decomposition of polymer mixtures of PVC, PET and ABS containing brominated flame retardant: Formation of chlorinated and brominated organic compounds. J. Anal. Appl. Pyrol..

[9] Altarawneh M., Saeed A., Al-Harahsheh M., Dlugogorski B.Z. (2019). Thermal decomposition of brominated flame retardants (BFRs): Products and mechanisms. Prog. Energ. Combust..

[10] Altarawneh M., Dlugogorski B.Z. (2015). Formation of polybrominated dibenzofurans from polybrominated biphenyls. Chemosphere.

[11] Blaiszik B., Sottos N., White S. (2008). Nanocapsules for self-healing materials. Compos. Sci. Technol..

[12] Noisser T., Reichenauer G., Husing N. (2014). In Situ Modification of the silica backbone leading to highly porous monolithic hybrid organic–inorganic materials via ambient pressure drying. ACS Appl. Mater. Interfaces.

[13] Pourhashem S., Vaezi M.R., Rashidi A. (2017). Investigating the effect of SiO_2_-graphene oxide hybrid as inorganic nanofiller on corrosion protection properties of epoxy coatings. Surf. Coat. Technol..

[14] Yuan B., Hu Y., Chen X., Shi Y., Niu Y., Zhang Y., He S., Dai H. (2017). Dual modification of graphene by polymeric flame retardant and Ni(OH)_2_ nanosheets for improving flame retardancy of polypropylene. Compos. Part A-Appl. Sci. Manuf..

[15] Shi Y., Yu B., Duan L., Gui Z., Wang B., Hu Y., Yuen R.K.K. (2017). Graphitic carbon nitride/phosphorus-rich aluminum phosphinates hybrids as smoke suppressants and flame retardants for polystyrene. J. Hazard. Mater..

[16] Shi Y.Q., Yu B., Zheng Y.Y., Yang J., Duan Z.P., Hu Y. (2018). Design of reduced graphene oxide decorated with DOPO-phosphanomidate for enhanced fire safety of epoxy resin. J. Colloid Interf. Sci..

[17] Hapuarachchi T.D., Peijs T. (2010). Multiwalled carbon nanotubes and sepiolite nanoclays as flame retardants for polylactide and its natural fibre reinforced composites. Compos. Part A-Appl. Sci. Manuf..

[18] Wang W., Pan H., Shi Y., Pan Y., Yang W., Liew K.M., Song L., Hu Y. (2016). Fabrication of LDH nanosheets on β-FeOOH rods and applications for improving the fire safety of epoxy resin. Compos. Part A-Appl. Sci. Manuf..

[19] Chen X., Jiang Y., Jiao C. (2014). Smoke suppression properties of ferrite yellow on flame retardant thermoplastic polyurethane based on ammonium polyphosphate. J. Hazard. Mater..

[20] Wang W., Pan H., Shi Y., Yu B., Pan Y., Liew K.M., Song L., Hu Y. (2015). Sandwichlike coating consisting of alternating montmorillonite and β-FeOOH for reducing the fire hazard of flexible polyurethane foam. ACS Sustain. Chem. Eng..

[21] Pan H., Wang W., Pan Y., Song L., Hu Y., Liew K.M. (2014). Formation of layer-by-layer assembled titanate nanotubes filled coating on flexible polyurethane foam with improved flame retardant and smoke suppression properties. ACS Appl. Mater. Interfaces.

[22] Yu B., Shi Y., Yuan B., Qiu S., Xing W., Hu W., Song L., Lo S., Hu Y. (2015). Enhanced thermal and flame retardant properties of flame-retardant-wrapped graphene/epoxy resin nanocomposites. J. Mater. Chem. A.

[23] Wang W., Kan Y., Pan H., Pan Y., Li B., Liew K.M., Hu Y. (2017). Phosphorylated cellulose applied for the exfoliation of LDH: An advanced reinforcement for polyvinyl alcohol. Compos. Part A-Appl. Sci. Manuf..

[24] Zhou K., Gao R., Qian X. (2017). Self-assembly of exfoliated molybdenum disulfide (MoS_2_) nanosheets and layered double hydroxide (LDH): Towards reducing fire hazards of epoxy. J. Hazard. Mater..

[25] Wang W., Kan Y., Liu J., Liew K.M., Liu L., Hu Y. (2017). Self-assembly of zinc hydroxystannate on amorphous hydrous TiO_2_ solid sphere for enhancing fire safety of epoxy resin. J. Hazard. Mater..

[26] Wang W., Kan Y., Pan Y., Yuan Y., Liew K.M., Hu Y. (2017). Urchinlike shells of TiO_2_ hollow spheres for improving the fire safety of epoxy resin. Ind. Eng. Chem. Res..

[27] Gu H., Guo J., He Q., Tadakamalla S., Zhang X., Yan X., Huang Y., Colorado H.A., Wei S., Guo Z. (2013). Flame-retardant epoxy resin nanocomposites reinforced with polyaniline-stabilized silica nanoparticles. Ind. Eng. Chem. Res..

[28] Ran S., Guo Z., Han L., Fang Z. (2014). Effect of a lewis acid catalyst on the performance of HDPE/BFR/GNPs composites. Ind. Eng. Chem. Res..

[29] Li L.P., Zheng T., Xu L.N., Li Z., Sun L.D., Nie Z.X., Bai Y., Liu H.W. (2013). SnO_2_–ZnSn(OH)_6_: A novel binary affinity probe for global phosphopeptide detection. Chem. Commun..

[30] Carosio F., Alongi J., Malucelli G. (2012). Layer by layer ammonium polyphosphate-based coatings for flame retardancy of polyester–cotton blends. Carbohydr. Polym..

[31] Carosio F., Di Blasio A., Cuttica F., Alongi J., Frache A., Malucelli G. (2013). Flame retardancy of polyester fabrics treated by spray-assisted layer-by-layer silica architectures. Ind. Eng. Chem. Res..

[32] Wang W., Pan Y., Pan H., Yang W., Liew K.M., Song L., Hu Y. (2016). Synthesis and characterization of MnO_2_ nanosheets based multilayer coating and applications as a flame retardant for flexible polyurethane foam. Compos. Sci. Technol..

[33] Wang W., Kan Y., Yu B., Pan Y., Liew K.M., Song L., Hu Y. (2017). Synthesis of MnO_2_ nanoparticles with different morphologies and application for improving the fire safety of epoxy. Compos. Part A-Appl. Sci. Manuf..

[34] Sinha A.K., Pradhan M., Pal T. (2013). Morphological evolution of two-dimensional MnO_2_ nanosheets and their shape transformation to one-dimensional ultralong MnO_2_ nanowires for robust catalytic activity. J. Phys. Chem. C.

[35] Wrobel G., Piech M., Dardona S., Ding Y., Gao P.X. (2009). Seedless synthesis and thermal decomposition of single crystalline zinc hydroxystannate cubes. Cryst. Growth Des..

[36] Ma Z., Shao G., Fan Y., Wang G., Song J., Shen D. (2016). Construction of hierarchical α-MnO_2_ nanowires@ ultrathin δ-MnO_2_ nanosheets core–shell nanostructure with excellent cycling stability for high-power asymmetric supercapacitor electrodes. ACS Appl. Mater. Interfaces.

[37] Fu X., Huang D., Qin Y., Li L., Jiang X., Chen S. (2014). Effects of preparation method on the microstructure and photocatalytic performance of ZnSn(OH)_6_. Appl. Catal. B-Environ..

[38] Luo P., Zhang H., Liu L., Fang L., Wang Y. (2016). Sandwich-like nanostructure of amorphous ZnSnO_3_ encapsulated in carbon nanosheets for enhanced lithium storage. Electrochim. Acta.

[39] Singu B.S., Hong S.E., Yoon K.R. (2017). Ultra-thin and ultra-long α-MnO_2_ nanowires for pseudocapacitor material. J. Solid State Electr..

[40] Li D., Li W., Deng Y., Wu X., Han N., Chen Y. (2016). Effective Ti doping of δ-MnO_2_ via anion route for highly active catalytic combustion of benzene. J. Phys. Chem. C.

[41] Wang W., Pan H., Yu B., Pan Y., Song L., Liew K.M., Hu Y. (2015). Fabrication of carbon black coated flexible polyurethane foam for significantly improved fire safety. RSC Adv..

[42] Miran H.A., Altarawneh M., Jiang Z.T., Oskierski H., Almatarneh M., Dlugogorski B.Z. (2017). Decomposition of selected chlorinated volatile organic compounds by ceria (CeO_2_). Catal. Sci. Technol..

