# Erratum: Electrostatic-Interaction-Driven Assembly of Binary Hybrids towards Fire-Safe Epoxy Resin Nanocomposites. *Polymers* 2019, 11, 229.

**DOI:** 10.3390/polym11040724

**Published:** 2019-04-19

**Authors:** Lu Liu, Wei Wang, Yongqian Shi, Libi Fu, Lulu Xu, Bin Yu

**Affiliations:** 1 College of Environment and Resources, Fuzhou University, 2 Xueyuan Road, Fuzhou 350116, China; lyqian@mail.ustc.edu.cn; 2 Hefei Institute for Public Safety Research, Tsinghua University, 5999 Xiyou Road, Hefei 230026, China; 3 State Key Laboratory of Fire Science, University of Science and Technology of China, 96 Jinzhai Road, Hefei 230026, China; wwei433@mail.ustc.edu; 4 Department of Architecture and Civil Engineering, City University of Hong Kong, Tat Chee Avenue, Kowloon 999077, Hong Kong; 5 College of Civil Engineering, Fuzhou University, 2 Xueyuan Road, Fuzhou 350116, China; fulibi@fzu.edu.cn; 6 School of Materials Science & Engineering, Nanyang Technological University, Singapore 639798, Singapore; lulu.xu@ntu.edu.sg

The authors wish to make a change to the published paper [1]. In the original manuscript, Figure 7a,b have been published in the previous work [2]. The corrected Figure 7 is presented below.

The authors apologize for any inconvenience caused and the change does not affect the scientific results. The manuscript will be updated and the original will remain online on the article webpage https://www.mdpi.com/2073-4360/11/2/229.

## Figures and Tables

**Figure 7:**
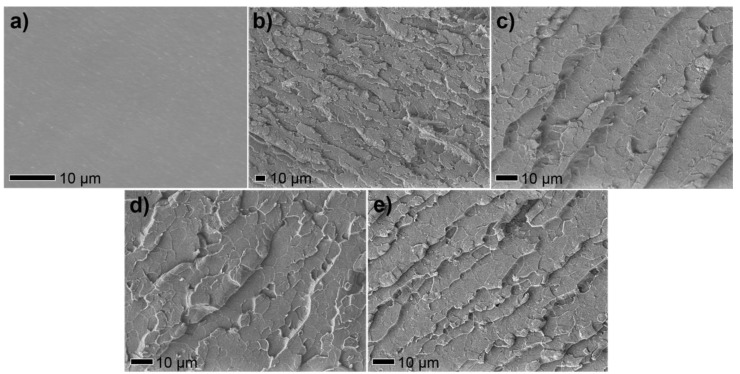
SEM images of fracture surfaces cryogenically broken after immersion in liquid nitrogen of (**a**) pure EP, (**b**) EP/MnO_2_ 2%, (**c**) EP/MnO_2_@ZHS 0.5%, (**d**) EP/MnO_2_@ZHS 1%, and** e)** EP/MnO_2_@ZHS 2%.
